# Analysis of Negative Results of Metagenomics Next-Generation Sequencing in Clinical Practice

**DOI:** 10.3389/fcimb.2022.892076

**Published:** 2022-05-16

**Authors:** Mengjia Qian, Bijun Zhu, Yanxia Zhan, Lingyan Wang, Qi Shen, Miaomiao Zhang, Lei Yue, Duojiao Wu, Hao Chen, Xiangdong Wang, Yunfeng Cheng

**Affiliations:** ^1^ Institute of Clinical Science, Zhongshan Hospital, Fudan University, Shanghai, China; ^2^ Department of Hematology, Zhongshan Hospital, Fudan University, Shanghai, China; ^3^ Center for Tumor Diagnosis & Therapy, Jinshan Hospital, Fudan University, Shanghai, China; ^4^ Department of Thoracic Surgery, Zhongshan Hospital Xuhui Branch, Fudan University, Shanghai, China; ^5^ Department of Hematology, Zhongshan Hospital Qingpu Branch, Fudan University, Shanghai, China

**Keywords:** metagenomics next-generation sequencing (mNGS), false negative, clinical practice, true negative, sample type

## Abstract

**Background:**

Metagenomics next-generation sequencing (mNGS) has been increasingly used in the clinic, which provides a powerful tool for the etiological diagnosis of infectious diseases. Precise treatment can be carried out according to the positive mNGS results. However, the role of negative results of mNGS remains poorly defined in clinical practice.

**Methods:**

The results of 1,021 samples from patients who received the mNGS test at Zhongshan Hospital, Fudan University, between January 2019 and December 2019 were analyzed.

**Results:**

There were 308 samples (30.17%) of negative results included in the current study. The top 2 types of negative samples were blood (130/308) and tissue (63/308), which also accounted for the highest negative proportion in diseases. Sputum and bronchoalveolar lavage fluid (BALF) were more likely to have positive results. In false-negative results (defined as negative in mNGS test but reported positive in other sample types or assays), 118 samples were found when compared to regular microbiological assays. The negative predictive value (NPV) of mNGS was 95.79% [95%CI, 93.8%–97.8%] as compared to culture and smear. *Mycobacterium*, *Aspergillus*, and *Mycoplasma* ranked as the top 3 microorganisms on the undetected pathogen list.

**Conclusions:**

The present data indicate that when the mNGS test is negative, the negative prediction accuracy rate of the original specimen is significant. However, other laboratory assays results and clinical presentations should always be carefully considered prior to drawing a diagnosis.

## Introduction

Infectious diseases are caused by a variety of pathogens, which lead to systemic or local inflammation (Honda and Littman, 2012) with significant mortalities (Lozano et al., 2012). As the clinical manifestations of infectious diseases are diverse, an accurate clinical diagnosis is challenging when a pathogen is unknown (Simner et al., 2018).

With the development of gene detection technology, metagenomics next-generation sequencing (mNGS) has been increasingly used in the clinic, which provides a powerful tool for the etiological diagnosis of infectious diseases (Chiu and Miller, 2019). As it directly extracts all microbial nucleic acids from clinical samples, mNGS theoretically meets the detection needs of all microorganisms in one detection (Goldberg et al., 2015; Li et al., 2021). Through unbiased and full coverage sequencing technology, the acquired sequence information is compared with a microbial database to obtain the specific species and relative content of microorganisms (Miao et al., 2018). Compared with the conventional culture assay, mNGS is less time-consuming, not limited by culture conditions, and capable of detecting those pathogens that are difficult to culture (Kitsios et al., 2018).

When the mNGS test is positive, precise treatment could be carried out soundly according to the actual clinical situation. However, the role of negative results of mNGS remains poorly defined in clinical practice. As such, the present study is aimed to evaluate the clinical value of negative mNGS results and to provide evidence for its proper applications.

## Methods

### Study Subjects

A total of 1,021 samples from hospitalized patients who received mNGS test at Zhongshan Hospital, Fudan University, from January 2019 to December 2019 were collected and reviewed. Results of regular clinical assays of these samples were also collected at the same time. The study was approved by the institutional review board of Zhongshan Hospital, Fudan University (#B2021-694R). Written informed consent was obtained from each patient prior to the enrollment.

### Metagenomics Next-Generation Sequencing Test

Samples were collected at standard procedures according to their various types including blood, sputum, bronchoalveolar lavage fluid (BALF), and tissue. Nucleic acid was extracted and fragmented into DNA fragments, and then these fragments were end-repaired, barcoded, and amplified to be qualified libraries. Libraries were sequenced on the BGISEQ-50 platform. Sequencing data were compared to human references, then human sequence reads were removed, and nonhuman sequence reads were further filtered and compared to databases (bacteria, fungi, and parasite databases). Final data including stringent mapped reads number (SMRN), coverage rate (CovRate), relative abundance (Re_Abu), and sequencing depth were analyzed and reported.

### Statistical Analysis

Continuous data are shown as mean values ± SE. Student’s t-test was used to measure the differences in variables, where appropriate. Data analysis was performed using SPSS 16.0 software. *p*-Values <0.05 were considered significant, and all tests were two-tailed.

## Results

### Sample Characteristics

**Figure 1:**
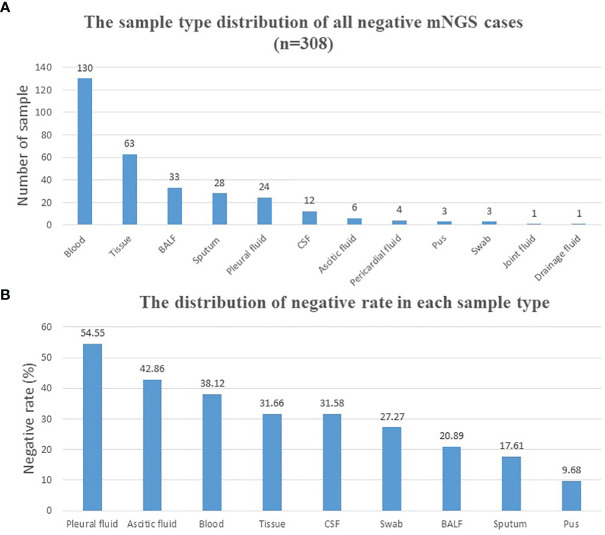
Distribution of negative mNGS cases. **(A)** Distribution of all mNGS negative cases according to different sample types. **(B)** Negative rate calculation and distribution of each sample type. BALF, bronchoalveolar lavage fluid; CSF, cerebrospinal fluid; mNGS, metagenomics next-generation sequencing.

A total of 1,021 samples were included in the current study, with 713 samples (69.83%) of positive results and 308 samples (30.17%) of negative results (**Table 1**). Reports that only with colonization or background microorganisms were considered negative. The sample type distribution of all negative samples is listed in **Figure 1A**. The most common negative sample type was blood (130/308, 42.2%). Tissue, including lung tissue, bone tissue, lymph node tissue, valve tissue, and skin tissue, ranked second (63/308, 20.5%). Other sample types included BALF, sputum, pleural fluid, cerebrospinal fluid (CSF), ascetic fluid, pericardial fluid, pus, swab, joint fluid, and drainage fluid. The negative rate was calculated in each sample type (sample numbers >10) and shown in **Figure 1B**. Pleural fluid (54.55%), ascetic fluid (42.86%), and blood (38.12%) ranked the top 3, and pus (9.68%) ranked the last.

**Table 1:** Sample characteristics.

Sample type	Negative mNGS cases	Positive mNGS cases
Blood	130 (42.2%)	211 (29.6%)
Tissue	63 (20.45%)	136 (19.1%)
BALF	33 (10.7%)	125 (17.5%)
Sputum	28 (9%)	131 (18.4%)
Pleural fluid	24 (7.79%)	20 (2.8%)
CSF	12 (3.9%)	26 (3.6%)
Ascitic fluid	6 (1.95%)	8 (1.12%)
Pericardial fluid	4 (1.3%)	3 (0.42%)
Pus	3 (1%)	28 (3.92%)
Swab	3 (1%)	8 (1.12%)
Joint fluid	1 (0.3%)	0 (0.00%)
Drainage fluid	1 (0.3%)	3 (0.42%)
Bile	0 (0.00%)	5 (0.7%)
Stool	0 (0.00%)	1 (0.14%)
Urine	0 (0.00%)	8 (1.12%)
Total	308	713

mNGS, metagenomics next-generation sequencing; BALF, bronchoalveolar lavage fluid; CSF, cerebrospinal fluid.

**Figure 2:**
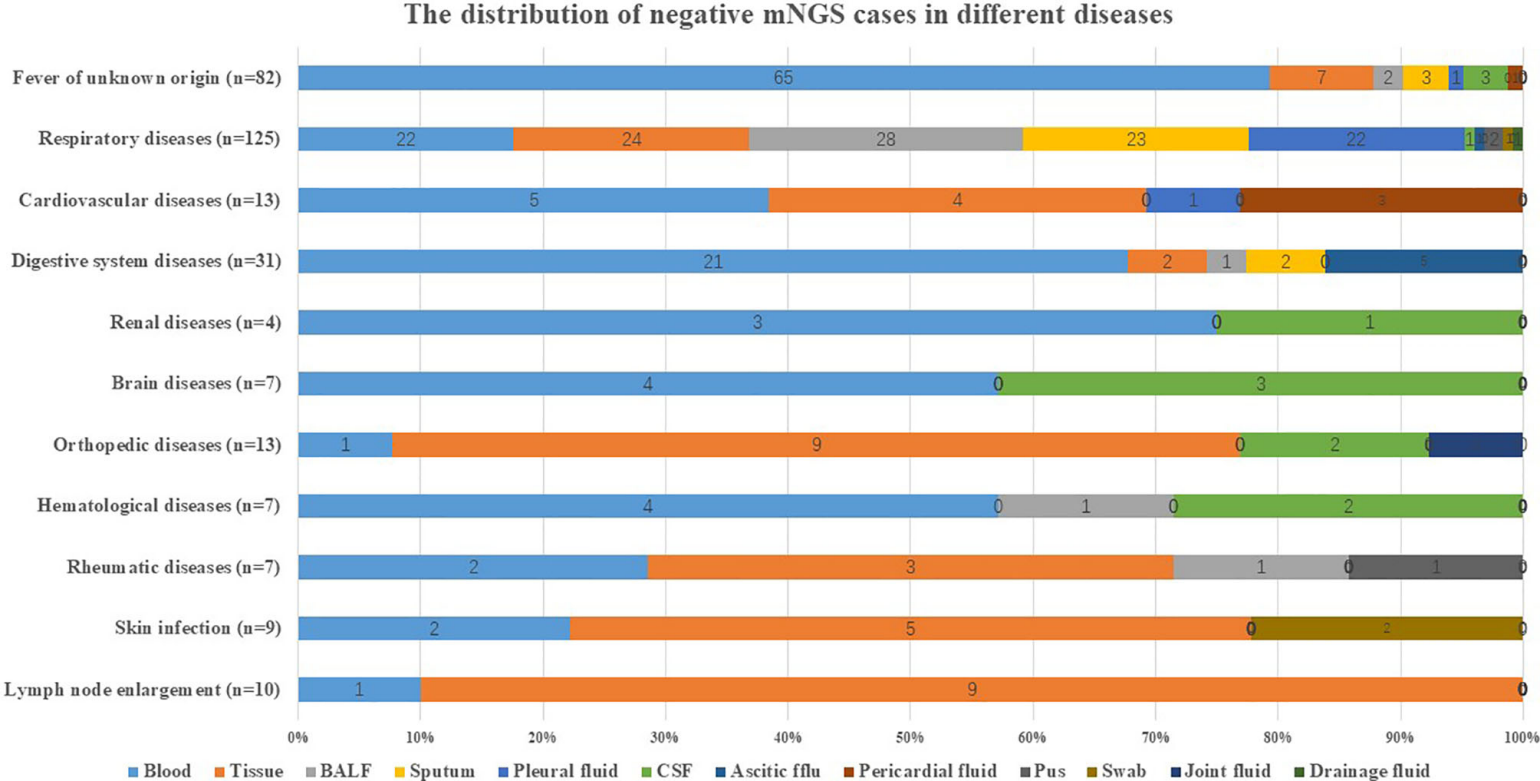
Distribution of negative mNGS cases in diseases. The distribution of negative mNGS cases in different diseases including fever of unknown origin, respiratory diseases, cardiovascular diseases, digestive system diseases, renal diseases, brain diseases, orthopedic diseases, hematological diseases, rheumatic diseases, skin infection, and lymph node enlargement. BALF, bronchoalveolar lavage fluid; CSF, cerebrospinal fluid; mNGS, metagenomics next-generation sequencing.

Blood and tissue accounted for the highest negative proportion in different common infectious diseases as listed in **Figure 2**. For instance, blood accounted for 79.27% (65/82) in a fever of unknown origin and 67.74% (21/31) in digestive system diseases. Tissue accounted for 90% (9/10) in lymph node enlargement and 69.23% (9/13) in orthopedic diseases.

### Concordance of Negative Result Between Metagenomics Next-Generation Sequencing and Conventional Assays

In order to study the influence of sample type on mNGS results, true negative (TN) and false negative (FN) were defined. Laboratory results that were negative in all sample types and all assays were defined as TN, and laboratory results that were positive in some sample types or some assays were defined as FN. Conventional assays included in our study were culture, smear, TSPOT.TB (TSPOT), cryptococcal antigen (CrAg), immunoassay, 1-3-β-d-glucan test (G test), and galactomannan test (GM test). In addition, parasite detection was all negative in all samples suspected of parasite infection, so it was not listed. All the results of other conventional assays were collected and sampled at the same time as mNGS. However, the paired-culture test was the basic premise of the comparison. Therefore, 23 cases were excluded because they had no paired-culture results. The remaining 285 cases were further classified in **Table 2** according to TN and FN. Common colonization pathogens detected in conventional assays were also considered to be negative results.

**Table 2:** Classification of true negative and false negative.

	Definition	Number of cases
**True negative**	Laboratory negative in all sample types and all assays	167 (58.6%)
**False negative**	Laboratory positive in some sample types or some assays	118 (41.4%)

According to the definition and statistical results, TN was 58.6% (167/285) and FN was 41.4% (118/285). FN positively differed among culture and smear in the same specimen, culture, smear, and mNGS of other sample types and other assays as listed in **Table 3**. One case might have several positive results.

**Table 3:** Classification of false-negative cases.

False-negative classification	Cases
**Culture and smear of same specimen**	
Culture positive in the same specimen	12
**Other sample types**	
Culture positive in other sample types	26
Smear positive in other sample types	1
mNGS positive in other sample types	22
**Other assays**	
TSPOT	48
CrAg	3
Immunoassay	25
G test	2
GM test	8

mNGS, metagenomics next-generation sequencing; TSPOT, TSPOT.TB; CrAg, cryptococcal antigen; G test, 1-3-β-d-glucan test; GM test, galactomannan test.

### Negative Predictive Value of Metagenomics Next-Generation Sequencing

**Figure 3:**
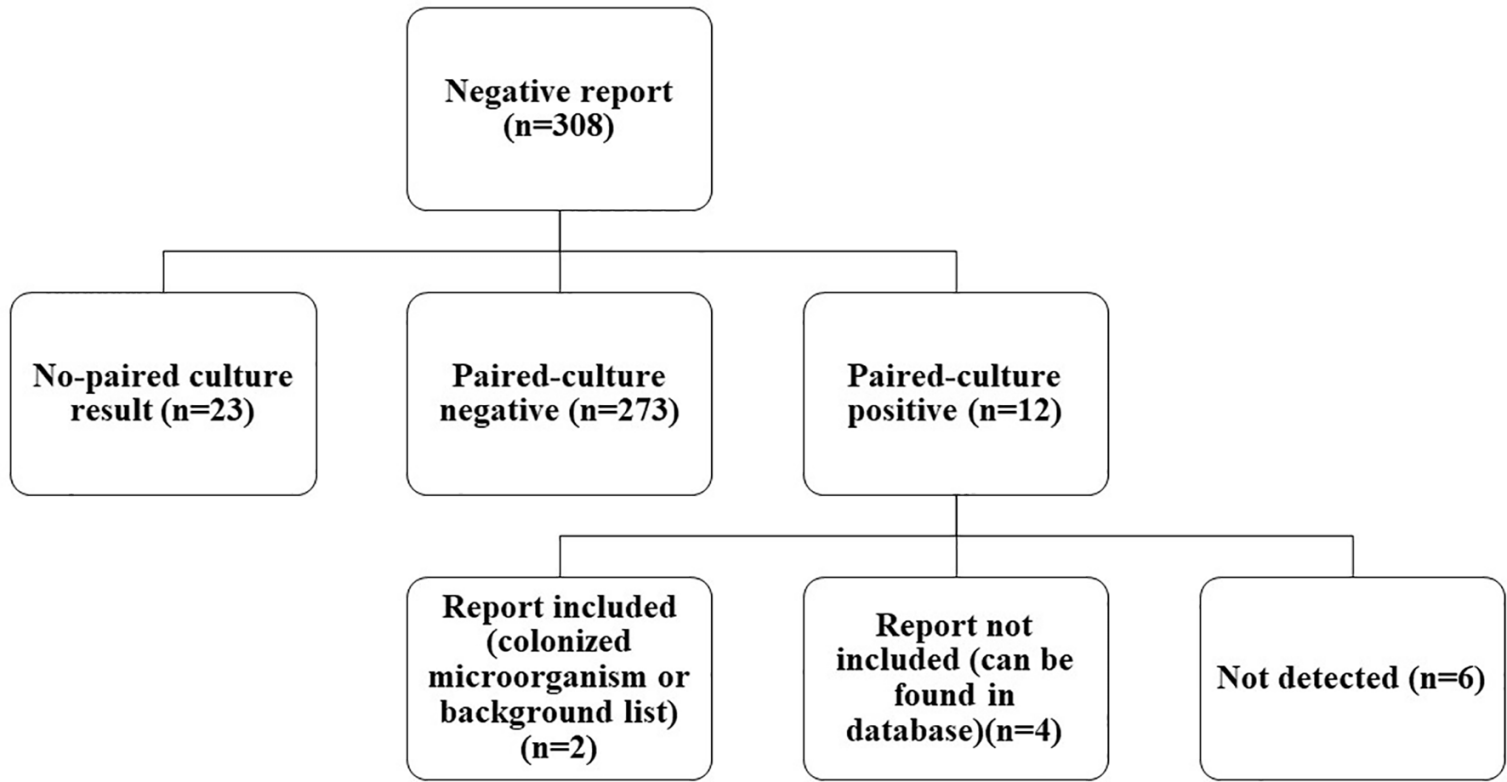
Classification of negative mNGS cases. In total, 308 negative mNGS cases were divided into no paired-culture result, paired-culture negative, and paired-culture positive. Twelve paired-culture positive cases were further divided into report included, report not included, and not detected. mNGS, metagenomics next-generation sequencing.

Among FN cases, there were 12 paired-culture positive cases (**Figure 3**). As compared with culture, which is the gold standard in pathogen detection, the negative predictive value (NPV) of mNGS was 273/285 = 95.79% [95%CI, 93.8%–97.8%]. With the removal of the six cases that were detected in a database or shown in the colonization (pathogenicity is not considered even if SMRN is high) or background (common contaminating pathogens in the laboratory) list, the NPV of mNGS could be increased to 279/285 = 97.89% (95%CI, 95.9%–99.8%), as detailed in **Table 4**. Among them, *Pseudomonas aeruginosa* detected in sample 21 was in the colonized microorganism list, and *Staphylococcus epidermidis* detected in sample 203 was in the background list. *Staphylococcus aureus* in sample 38, *P. aeruginosa* in sample 118, *Staphylococcus hominis* in sample 153, and *Actinomyces* in sample 178 were not shown in the report but could be found in the database.

**Table 4:** List of 12 paired-culture positive cases.

		Culture result	mNGS result					
Sample ID	Type	Culture result	CovRate	Depth	Re_Abu	Genus_Re_Abu	SMRN	SMRNG
21	BALF	*Pseudomonas aeruginosa*	0.2029	1	2.59	37.36	232	1855
26	Tissue	*Mycobacterium tuberculosis*	0	0	0	0	0	0
35	BALF	*M. tuberculosis*	0	0	0	0	0	0
38	Sputum	*Staphylococcus aureus*	0.0017	1	0.01	0.07	1	1
118	BALF	*P. aeruginosa*	0.0081	1	0.46	0.9	10	14
120	Drainage fluid	*P. aeruginosa*	0	0	0	0	0	0
153	Pleural fluid	*Staphylococcus hominis*	0.0044	1	0.58	1.6	1	3
178	Tissue	*Actinomyces*	0.0016	1		0.31		2
186	Ascitic fluid	*M. tuberculosis*	0	0	0	0	0	0
203	Pleural fluid	*Staphylococcus epidermidis*	0.0019	1	1.51	6.44	1	1
248	Blood	*Cryptococcus humicolus*	0	0	0	0	0	0
266	Tissue	*Corynebacterium afermentans*	0	0	0	0	0	0

mNGS, metagenomics next-generation sequencing; BALF, bronchoalveolar lavage fluid; CovRate, coverage rate; Re_Abu, relative abundance; SMRN, stringent mapped reads number at species level; SMRNG, stringent mapped reads number at genus level.

### Impact of Sample Type and Distribution of Undetected Pathogens

**Figure 4:**
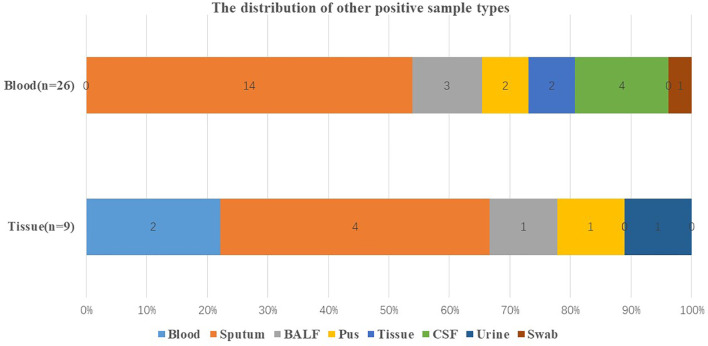
Distribution of other positive sample types. Distribution of other positive sample types when the original sample type was blood and tissue that were with high negative rate. BALF, bronchoalveolar lavage fluid; CSF, cerebrospinal fluid.

As shown in **Table 3**, there were 26 culture positive cases, 1 smear positive case, and 22 mNGS positive cases in other sample types. In terms of blood and tissue that had high negative rates, respiratory samples including sputum and BALF were more likely to have positive results (**Figure 4**).

**Figure 5:**
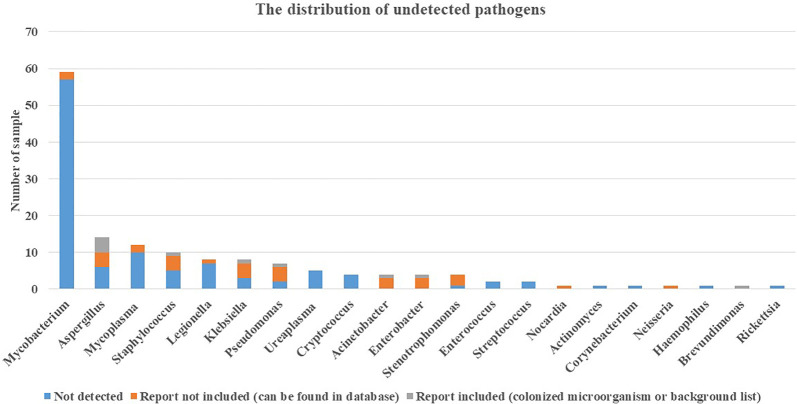
Distribution of undetected pathogens. Pathogens detected positive in assays include culture, mNGS (other sample types), TSPOT, CrAg, immunoassay, G test, and GM test were further classified in no sequence detected in mNGS, report included, and report not included. mNGS, metagenomics next-generation sequencing; TSPOT, TSPOT.TB; CrAg, cryptococcal antigen; G test, 1-3-β-D-glucan test; GM test, galactomannan test.

In addition to culture, other assays commonly used in clinics include TSPOT for *Mycobacterium tuberculosis*, CrAg for *Cryptococcus*, G test and GM test for fungus, and immunoassay for mycoplasma, *Legionella*, etc. There were 48 cases that were positive in the TSPOT test, but in the mNGS test, no *Mycobacterium* was detected in these cases, even in the database. G test and GM test were effectively used to detect *Aspergillus* (Li et al., 2019), and immunoassay was effective for *Mycoplasma* detection (Parker et al., 2018). These three pathogens were in the top 3 in the undetected pathogen list shown in **Figure 5**. In addition, there were 3 cases reported positive in CrAg and 1 case reported *Cryptococcus* positive in blood culture. Furthermore, there was a case of *Rickettsia* detection in immunoassay, and no sequence was detected in mNGS.

### Comparison of Experimental Data and Infection Indexes Between True-Negative and False-Negative Samples

**Figure 6:**
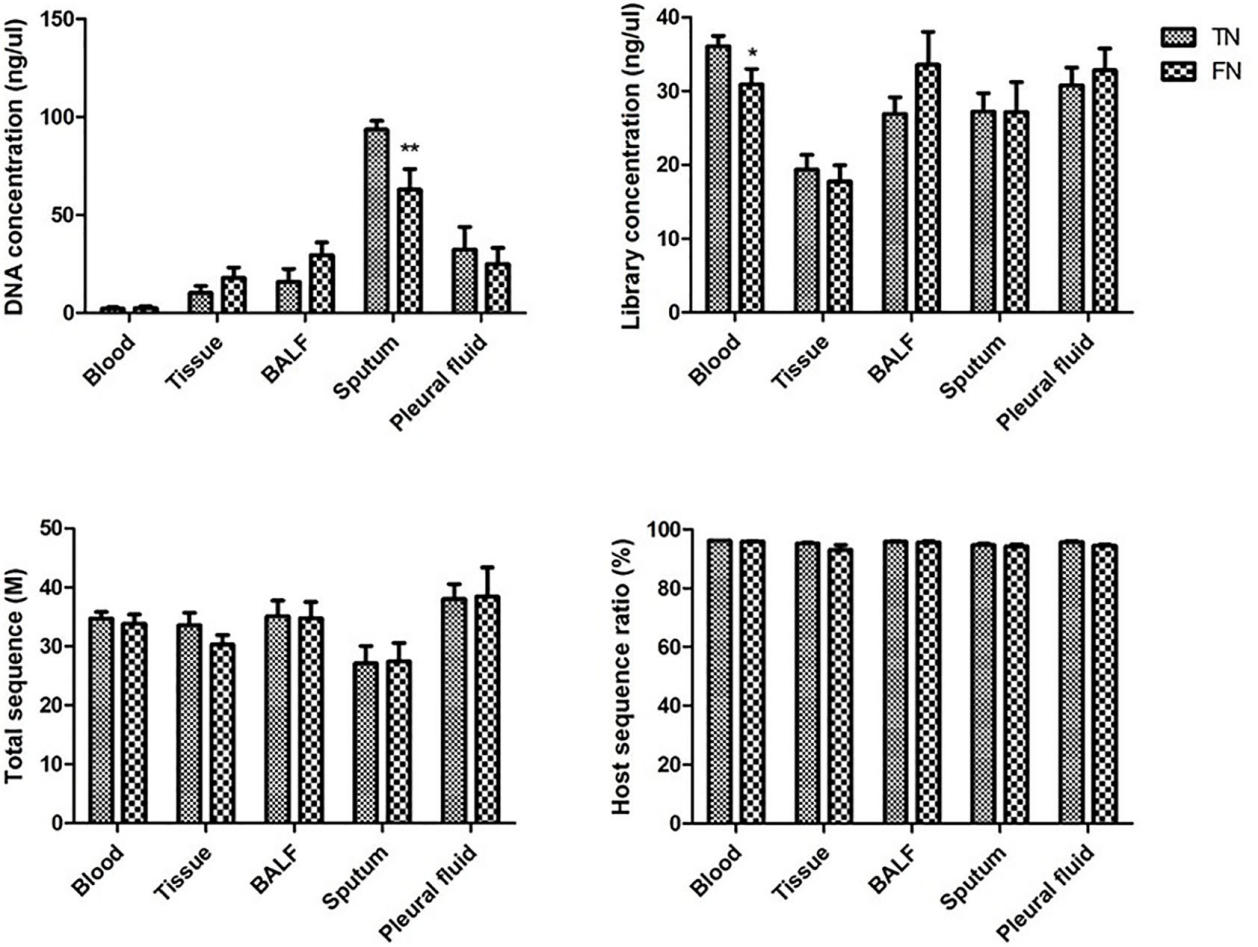
Comparison of experimental data between TN and FN samples. DNA concentration, library concentration, total sequence, and host sequence ratio were compared between TN and FN samples in blood, tissue, BALF, sputum, and pleural fluid. *p < 0.05, statistical difference. **p < 0.01, significant statistical difference. BALF, bronchoalveolar lavage fluid; TN, true negative; FN, false negative.

In order to further exclude the influence of FN from the laboratory, we collected the experimental data of all the negative samples. The data collected cover the whole process of mNGS, including DNA concentration, library concentration, total sequence, and host sequence ratio. Bur for some sample types, since the total amount of the sample type is small, it is not statistically significant and is not included. The comparison of blood, tissue, BALF, sputum, and pleural fluid is shown in **Figure 6**. In the DNA extraction step, DNA concentration was significantly lower in FN samples than in TN samples in sputum. In the library construction step, library concentration was lower in FN samples than in TN samples in blood. However, in the last laboratory step, sequencing, neither the total sequence nor the host sequence ratio had a significant difference between TN and FN.

**Figure 7:**
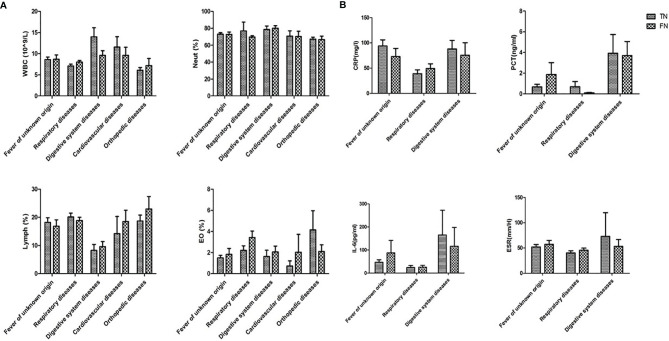
Comparison of infection indexes between TN and FN samples. **(A)** WBC, Neut, Lymph, EC, **(B)** CRP, PCT, IL-6, ESR were compared between TN and FN samples in different diseases. WBC, leukocyte; Neut, neutrophil; Lymph, lymphocyte; EO, eosinophilia; CRP, C-reactive protein; PCT, procalcitonin; IL-6, interleukin-6; ESR, erythrocyte sedimentation rate; TN, true negative; FN, false negative.

In addition, infection indexes include leukocyte (WBC), neutrophil (Neut), lymphocyte (Lymph), eosinophilia (EO), C-reactive protein (CRP), procalcitonin (PCT), interleukin-6 (IL-6), and erythrocyte sedimentation rate (ESR) of all the negative samples were collected. The disease groups with insufficient data for statistical analysis were removed. The comparison of WBC, Neut, Lymph, and EO between TN and FN is shown in **Figure 7A**, and CRP, PCT, IL-6, and ESR are shown in **Figure 7B**. No significant difference was observed between TN and FN.

## Discussion

In the present study, 1,021 mNGS reports were analyzed to evaluate the role of mNGS in clinical practice, in particular when the results were negative. The overall negative rate was 30.17%. Blood and tissue had the most frequent negative reports, accounting for 42.21% and 20.45%, respectively. According to the negative rate of all samples, blood (38.12%) and tissue (31.66%) also accounted for the third and fourth place of the negative rate. In addition, according to the distribution of diseases, the negative proportion of blood and tissue in various diseases was greater than that of other sample types.

Although the negative result rates of blood and tissue were high, compared with culture and smear, the NPV of mNGS reached 95.79% (95%CI, 93.8%–97.8%) in the current study, which implies that when a negative mNGS result is reported, the negative prediction accuracy rate of the original specimen is high. The NPV of another study was 100% (95%CI, 71.7%–100%) for bacteria, 72.7% (95%CI, 39.3%–92.7%) for fungi, and 100% (95%CI, 76%–100%) for *M. tuberculosis* in mNGS vs. culture and smear (Li et al., 2018). However, when other assays, such as TSPOT, were added to the NPV comparison, the proportion of undetected *M. tuberculosis* increased significantly. In our study, only 2 cases were cultured *M. tuberculosis* positive in the same specimen, 2 cases were culture positive, and 1 case was smear positive in other sample types; however, 48 cases were TSPOT positive. A positive TSPOT may merely due to the manifestation of old tuberculosis (Ma et al., 2019), especially when its mNGS test is negative. Hence, it is recommended that when a specific infection is suspected in the clinic, other proper assays should also be used as combined diagnostic measures.

Some pathogens with low SMRN or SMRNG were excluded in the report, and some pathogens were identified as colonized or background microorganisms. When the report is negative and there is a suspected specific pathogen infection in the clinic, it can be traced back to the original database for a query.

The causes of FN both in the clinic and in the laboratory were analyzed. The first is the selection of sample types in the clinic. Clinicians should have a comprehensive consideration of the sample type for the mNGS test including the clinical symptoms, the location, and the type of the infection (Liu et al., 2019). However, blood and tissue samples should be avoided if there are other sample types available for selection. If there are indications of respiratory tract infection, we recommend respiratory tract samples for the mNGS test. The negative rates of sputum (17.61%) and BALF (20.89%) were much lower than those of other sample types in our study. Moreover, the false-negative samples of blood and tissue were reanalyzed, and the data showed that when both culture and mNGS were negative when sputum and BALF samples were sent for detection, the positive rate would be significantly increased. Furthermore, in the case of unknown pathogen infection, selecting multiple sample types for combined detection is recommended (Wang et al., 2020).

Second, the correlation between the status of infection and FN was explored. Complete blood count, CRP, PCT, IL-6, and ESR were collected on the day of examination of mNGS to determine the differences between FN and TN. There were no statistical differences between these two groups, which was consistent with another study (Duan et al., 2021). The data of the infection indexes showed that the samples with a clinical diagnosis of respiratory disease tended to be non-infectious diseases in mNGS samples. Therefore, the differential diagnosis (tumor, autoimmune diseases, etc.) should be considered in the clinical practice (Consensus Group Of Experts On Application Of Metagenomic Next Generation Sequencing In The Pathogen Diagnosis In Clinical M, Severe I, Professional Committee Of S, Shock Chinese Research Hospital A, Professional Committee Of Microbial Toxins Chinese Society For M, Professional Committee Of Critical Care Medicine Shenzhen Medical A, 2020).

Third, the experimental data were compared to analyze the false-negative results in the experiment. There were differences between FN and TN groups in DNA concentration of sputum and library concentration of blood. When there is lower DNA concentration in sputum samples and lower library concentration in blood emerged, we should be vigilant. The experimental steps should first make sure that each step is double-checked. When it turned into a negative report, it should be returned to the clinic, and clinicians should be reminded to make a comprehensive diagnosis and judgment. There were no statistical differences between FN and TN groups in the sequencing step. Therefore, increasing the amount of data is meaningless for false-negative samples. Moreover, the host sequence ratio had no reference significance in the judgment of false negatives.

Furthermore, *Mycobacterium* and *Aspergillus* were found to be the two most undetected pathogens. A possible explanation is that the cell walls of these pathogens were both thick and elaborate (Alderwick et al., 2015; Gow et al., 2017). It implies that before DNA extraction, precaution should be observed for the step of cell wall broken to obtain optical results.

Some limitations exist in our study. First, the definition of negative and positive in the study is limited to laboratory results, without clinical verification. Second, the laboratory results of bacteria, fungi, and parasites were analyzed in this study. Although viruses can be divided into DNA viruses and RNA viruses, the mNGS test in the current study only collected the result of DNA, as RNA viruses would degrade and could not be detected (Chen et al., 2020). Therefore, virus research was not included in the present study. Third, since prior antibiotic exposure has been reported to have less impact on the results of the mNGS test, the impact of antibiotics was not examined in study (Miao et al., 2018).

In conclusion, the present study showed that when the mNGS result is negative, the negative prediction accuracy rate of the original specimen is significant. However, other laboratory results and clinical presentations should always be carefully considered prior to the diagnosis.

## Data Availability Statement

The original contributions presented in the study are included in the article/supplementary material. Further inquiries can be directed to the corresponding authors.

## Ethics Statement

The studies involving human participants were reviewed and approved by Zhongshan Hospital, Fudan University. The patients/participants provided their written informed consent to participate in this study.

## Author Contributions

MQ, BZ, XW, and YC conceived the study. BZ, YZ, LW, QS, MZ, and LY performed the experiments and analyzed the data. MQ, DW, HC, XW, and YC performed the literature review and drafted and revised the manuscript. All authors read and approved the final manuscript.

## Funding

This work was supported by grants from the Program of Shanghai Academic/Technology Researcher leader (20XD1401000), Key Subject Construction Program of Shanghai Health Administrative Authority (ZK2019B30), the Shanghai Engineering Research Center of Tumor Multi-Target Gene Diagnosis (20DZ2254300), and Innovation Project funded by Science and Technology Commission of Jinshan District (2018-3-5). All authors obtained permission to acknowledge all those mentioned in the *Acknowledgments*.

## Conflict of Interest

The authors declare that the research was conducted in the absence of any commercial or financial relationships that could be construed as a potential conflict of interest.

## Publisher’s Note

All claims expressed in this article are solely those of the authors and do not necessarily represent those of their affiliated organizations, or those of the publisher, the editors and the reviewers. Any product that may be evaluated in this article, or claim that may be made by its manufacturer, is not guaranteed or endorsed by the publisher.
